# Supplementary material for: Transitioning between preparatory and precisely sequenced neuronal activity in production of a skilled behavior
Source: eLife. 2019 Jun 11;8:e43732. doi: 10.7554/eLife.43732 (PMC6592689; doi:10.7554/eLife.43732)
Supplement: Supplementary file 1. — *Male did not sing despite having a female present. **Male was actively calling during this trial. ***Male did not sing despite being in the presence of a female, however, the bird does perform introductory notes. [file elife-43732-supp1.docx]

**Supplementary file 1**

| **Bird** | **Trial** | **Type** | **Syllables** | **Motifs** | **Bouts** | **Phrases** | **Singing Duration (s)** |
| --- | --- | --- | --- | --- | --- | --- | --- |
| **O262** | 132226 | Directed | 30 | 6 | 3 | 1 | 4.92 |
|  | 135632 | Directed | 25 | 5 | 1 | 1 | 4.16 |
|  | 142315 | Directed | 15 | 3 | 1 | 1 | 2.43 |
|  | 173107 | Directed | 25 | 5 | 1 | 1 | 3.29 |
|  | 174745 | Directed | 25 | 5 | 2 | 1 | 4.08 |
|  | 165515 | Directed | 25 | 5 | 1 | 1 | 4.54 |
|  | 170812 | Non-Singing* | N/A | N/A | N/A | N/A | N/A |
| **O248** | 162048 | Directed | 244 | 49 | 24 | 10 | 31.73 |
|  | 162201 | Directed | 85 | 17 | 7 | 5 | 10.58 |
|  | 160046 | Directed | 170 | 34 | 21 | 4 | 22.92 |
|  | 152940 | Directed | 25 | 5 | 1 | 1 | 3.625 |
|  | 140745 | Undirected | 60 | 12 | 3 | 2 | 8.68 |
|  | 141006 | Undirected | 100 | 20 | 4 | 2 | 15.952 |
|  | 141059 | Undirected | 130 | 26 | 4 | 2 | 19.918 |
|  | 131612 | Undirected | 110 | 22 | 4 | 3 | 16.83 |
|  | 132211 | Non-Singing | N/A | N/A | N/A | N/A | N/A |
|  | 131904 | Non-Singing | N/A | N/A | N/A | N/A | N/A |
|  | 123106 | Non-Singing** | N/A | N/A | N/A | N/A | N/A |
| **O213** | 164915 | Directed | 30 | 7 | 4 | 1 | 5.1 |
|  | 170209 | Directed | 27 | 6 | 3 | 1 | 4.499 |
|  | 163439 | Directed | 27 | 6 | 3 | 1 | 4.55 |
|  | 165959 | Directed | 12 | 3 | 2 | 1 | 2.07 |
| **Y204** | 151708 | Directed | 30 | 9 | 2 | 1 | 5.61 |
|  | 155748 | Directed | 14 | 4 | 1 | 1 | 2.57 |
| **B89** | 162205 | Directed | 26 | 8 | 1 | 1 | 4.68 |
| **B263** | 163144 | Directed | 21 | 3 | 2 | 1 | 7.34 |
|  | 162125 | Directed | 14 | 2 | 1 | 1 | 2.72 |
|  | 140432 | Directed | 28 | 4 | 2 | 1 | 4.4 |
|  | 135424 | Non-Singing*** | N/A | N/A | N/A | N/A | N/A |
| **TOTALS** | 29 |  | 1298 | 266 | 98 | 45 | 197.194 |
