# Supplementary material for: Transitioning between preparatory and precisely sequenced neuronal activity in production of a skilled behavior
Source: eLife. 2019 Jun 11;8:e43732. doi: 10.7554/eLife.43732 (PMC6592689; doi:10.7554/eLife.43732)
Supplement: Supplementary file 2. [file elife-43732-supp2.docx]

**Supplementary file 2**

| Category of HVC_RA_ Neurons | Definition |
| --- | --- |
| Song Neurons | Active only during singing |
| Peri-Song Neurons | Active within the 5seconds before (-5.0 - 0s) or after singing (0 - +5.0s), but not during singing |
| Pan-Song Neurons | Active during singing and within the 5seconds before or after singing |
